# Effect of Yeast Cell Morphology, Cell Wall Physical Structure and Chemical Composition on Patulin Adsorption

**DOI:** 10.1371/journal.pone.0136045

**Published:** 2015-08-21

**Authors:** Ying Luo, Jianguo Wang, Bin Liu, Zhouli Wang, Yahong Yuan, Tianli Yue

**Affiliations:** College of Food Science and Engineering, Northwest A&F University, Yangling, Shaanxi, 712100, China; LAAS-CNRS, FRANCE

## Abstract

The capability of yeast to adsorb patulin in fruit juice can aid in substantially reducing the patulin toxic effect on human health. This study aimed to investigate the capability of yeast cell morphology and cell wall internal structure and composition to adsorb patulin. To compare different yeast cell morphologies, cell wall internal structure and composition, scanning electron microscope, transmission electron microscope and ion chromatography were used. The results indicated that patulin adsorption capability of yeast was influenced by cell surface areas, volume, and cell wall thickness, as well as 1,3-β-glucan content. Among these factors, cell wall thickness and 1,3-β-glucan content serve significant functions. The investigation revealed that patulin adsorption capability was mainly affected by the three-dimensional network structure of the cell wall composed of 1,3-β-glucan. Finally, patulin adsorption in commercial kiwi fruit juice was investigated, and the results indicated that yeast cells could adsorb patulin from commercial kiwi fruit juice efficiently. This study can potentially simulate *in vitro* cell walls to enhance patulin adsorption capability and successfully apply to fruit juice industry.

## Introduction

Patulin, which is mainly isolated from rotten fruits, may be introduced into fruit-based products during the industrial production. The presence of patulin in fruits has become a severe threat to food production and safety. Reports have implicated that patulin could induce a number of acute, chronic, and cellular-level health effects [1–3]. FAO/WHO established a provisional maximum daily intake of 0.4 μg/kg body weight for patulin because of its toxicity [4]. Patulin, which is once considered only exist in apple products, but now, its contamination in kiwi fruit have been reported [5, 6]. The quality of kiwi fruit products have also became an increased concern.

Physical and chemical methods have been developed for the removal of patulin [7–9]. However, most of these methods did not become popular because of their high cost or weak binding capability [10]. Biological adsorption has recently been considered the most effective strategy for the management of patulin in food industry [11–13]. Among the potential decontaminated microorganisms, yeast has unique advantages, such as easy cultivation, low cost, and being nonhazardous. Yeast cells could degrade patulin during fermentation. Stinson reported that the eight commercial yeast strains used in their study reduced the total patulin content by 99% or higher during yeast fermentation. Their names were Montrachet 522, Champagne, Burgundy 4123, California 4105, Muscatel 8256, Sauterne 8257, Steinberg 14284 and Wortman 4098, respectively [14]. In a study by Burroughs, approximately 90% of the patulin was removed during yeast fermentation [15]. Moreover, inactivated yeast cells also have high binding capability, and patulin adsorption is specifically strain specific according to Yue et al. [12]. This fact indicates that inactivated cells do not lose their patulin adsorption capability [16–18]. Based on these studies, the removal of patulin occurs in cells through cell surface rather than by metabolism.

Yeast cell wall consist about 20%-30% (w/w) of the total weight of a cell and has a bi-layered structure mainly composed of polysaccharides, particularly alkali-insoluble β-glucans, alkali-soluble β-glucans, mannan and minor chitin [19–21]. The inner layer of glucans (mainly 1,3-β-glucan) serving as a scaffold for the entire cell wall, is important for the cell wall 3D-network. This layer also protects the outer layer of mannoproteins, covalently linked to 1,3-β-glucans through 1,6-β-glucan chains. Although chitin has a minor content, it has an important role in cell wall structure. Chitin links 1,3-β-glucans through covalent bonds, resulting in their insolubility in alkali medium [22]. The 3D-network of the cell wall and the cell volume can be altered in response to osmotic challenges. Different yeast species present different cell surface properties and cell wall compositions. Rogers investigated the polysaccharide composition of the cell walls of several yeast species, such as *Debaryomyces hansenii*, *Zygosaccharomyces bailii*, *Saccharomyces cerevisiae*, et al. The results indicated that the cell wall composition varied over the species and strains [23].

Yeast cells are known to bind different molecules, such as mycotoxins and metal ions, through complex binding structures on the cell wall surface and the binding sites have been identified as cell wall polysaccharides [24]. Devegowda has shown the role of mannan from yeast cell wall in aflatoxin binding [25]. However, in a more recent work by Yiannikouris [26], it was demonstrated a predominant role of β-glucans to complex aflatoxin B1 (AFB1). It is also reinforced by the fact that AFB1 has an affinity of 72% for the mutant mnn9, as compared to 94.8% for the wild-type strain. This strain is deleted to MNN9 encoding a mannosyltransferase and exhibited a lower content of mannans accompanied to a higher content of β-glucans [21, 27–28]. The binding of patulin in regard to β-glucans conformation (both β-1,3 and β-1,6-glucans) was also investigated by Yiannikouris et al., it demonstrated the single helical conformation of 1,3-β-glucans played a major role on patulin complexation, and the stereochemistry and hydrophobic properties of toxin were important during β-D-glucans binding [26]. Studies on binding zearalenone and T-2 toxins with β-D-glucans were also discussed elsewhere [28–29]. Previous studies have shown that polysaccharides in the cell wall are closely associated with toxin adsorption. However, further studies on patulin adsorption from the microscopic viewpoint are needed.

To determine the mechanism of the patulin adsorption of yeast cells, the cell surface morphology and specific analysis for the cell wall of four different yeast strains were studied and performed, respectively. The objectives of this study were: (1) to determine whether patulin removal occurs through cell surface adsorption; (2) to identify the relationship of the cell surface properties and cell wall with patulin adsorption; (3) to verify how cell wall and alkali-insoluble 1,3-β-glucans affect the patulin adsorption capability; and (4) to attempt inactive yeast cells apply to commercial kiwi fruit juice.

## Materials and Methods

### Patulin solution preparation

Patulin (4-hydroxy-4H-furo [3,2c] pyran-2-[6H]-one), was purchased from sigma (purity > 99%). Standard stock solution of patulin at concentration of 100 mg/L was stored in ethyl acetate at -40°C. Patulin working solution was prepared by evaporating 2 mL of ethyl acetate stock solution at 40°C in a water bath. After that, patulin was dissolved in 1000 mL of 0.5% acetic acid solution (pH 4.0). A concentration of 200 μg/L patulin solution was prepared.

### Different forms of yeast cells preparation and patulin adsorption

**Fig 1:**
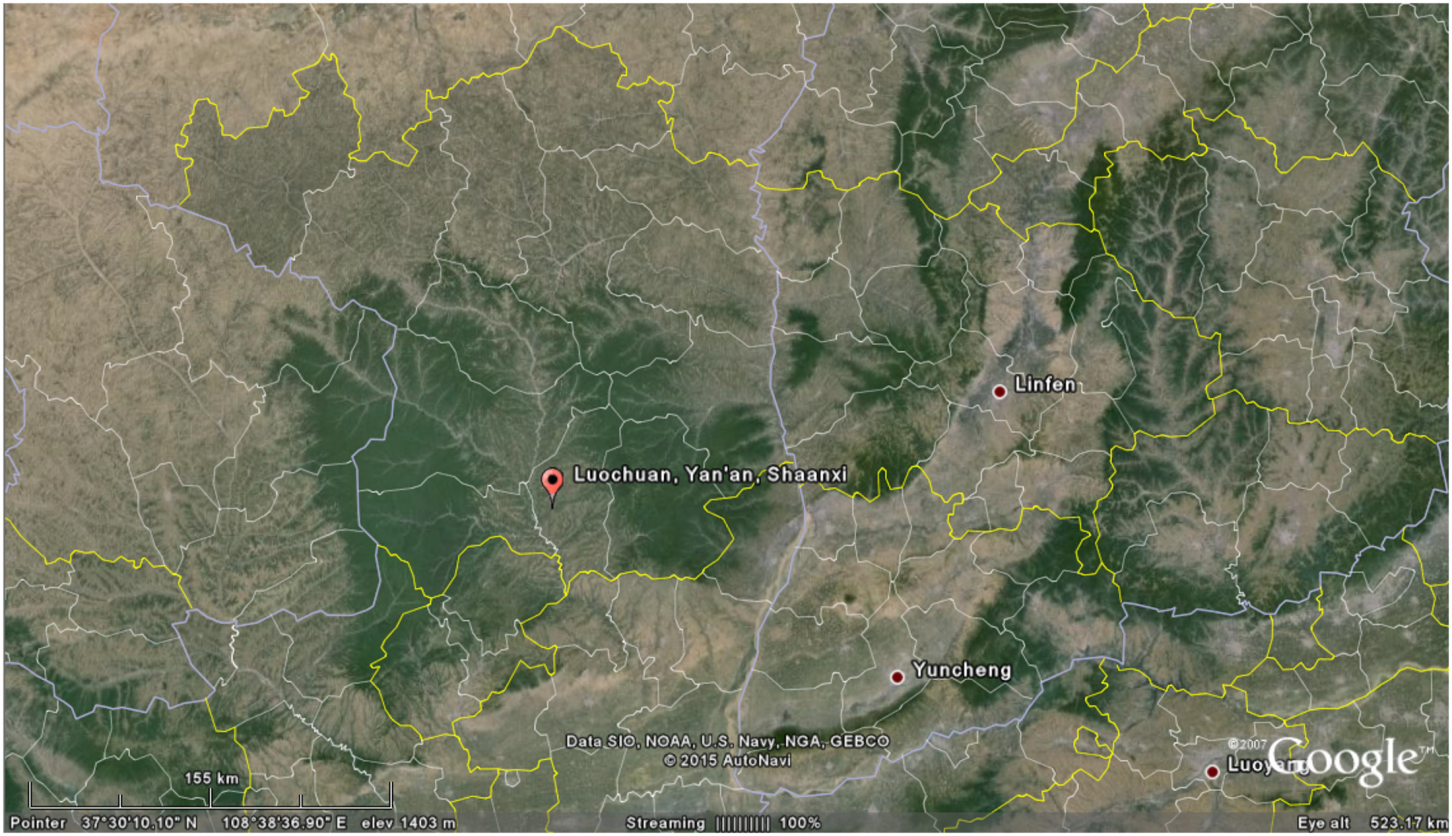
The geographic coordinate of the Luochuan orchard by google maps.

In the study, four yeast strains were used: *S*. *cerevisiae* 7# was purchased from China Center of Industrial Culture Collection (Beijing, China), *S*. *cerevisiae* WLS-38 was an electric fusion strain from our laboratory [30]. *Candida tropicalis* N-10 and *Pichia anomala* B-2p were wild types isolated from Luochuan orchards (Shaanxi, China) (Fig 1). The above strains were cultivated in yeast extract peptone dextrose medium (glucose 2%, peptone 2% and yeast extract powder 1%), and placed on a shaker incubator at 120 rpm, and 30°C for 24 h. After the enrichment incubation, the yeast cells (10^10^ CFU/mL) were collected through centrifugation at 3600 × *g* for 5 min, and washed twice with sterilized water.

To prepare inactivated cells, the cultured yeast cells were killed by autoclaving at 121°C for 10 min, cell viability was detected with methylene blue staining. The protoplasts were separated using snail enzyme (Biotopped, China) and by incubating for 3 to 6 h in citrate-phosphate buffer, pH 5.4 (2g Na_2_HPO_4_·12H_2_O and 0.47g C_6_H_18_O_7_·H_2_O dissolved in 100 mL distilled water), with 0.6 mol/L KCl and 0.01 mol/L MgSO_4_ [31]. To prepare damaged cells, the cultured yeast cells were suspended in 2 mL of absolute ethanol for 6 h at room temperature. All treated cells were collected and washed three times with sterilized water after reaction.

### Patulin adsorption assay and quantification

The different cell forms (3 × 10^6^ per milliliter) were suspended in 1 mL of 200 μg/L patulin solution and incubated for 20 h (150 rpm, at room temperature) in a shaker incubator. The control sample was without cells adding. Three replications were prepared for each sample, and the independent experiments were performed three times. After 20 h, yeast cells were removed through centrifugation at 3600 × *g* for 5 min, and then the supernatant was collected and analyzed.

Patulin in the supernatant was detected using high-performance liquid chromatography (Shimadzu Scientific Instruments, Columbia, MD) connected with a UV absorbance detector set at 276 nm [12]. Patulin was separated by a C18 reversed-phase column (250 × 4.6 mm, 5 μm diameter, Agilent Technologies, USA). Twenty (20) μL of the sample was injected [32], and an acetonitrile: water (10: 90) was used as isocratic mobile phase with a flow rate of 1 mL/min at 30°C, elution time was 15 min for each sample. The limit of detection was evaluated at 0.01 mg/L. The patulin adsorption efficiency (R %) was calculated using the following equation:

$$R\%=\frac{\left(C_{0}-C_{f}\right)}{C_{0}}\times 100$$ (1)

where *C*_*0*_ and *C*_*f*_ are the initial and final concentrations of patulin (mg/L), respectively.

### Cell morphology analysis

Scanning electron microscope (SEM) plays an important role in microbiology due to its high resolution. It has been a key provider of cellular micro-structure observation and analysis in microorganisms. In this study, the physical structure and morphology of the yeast cells were evaluated through SEM (S-3400, Hitachi Ltd. Japan). The length and width of each cell was measured using the SEM photographs. To calculate the cell surface area and volume, the yeast cells were assumed to be elliptical spheroids [33]. The structure of the spheroids were mainly formed with length (l) = 2 × major axis (a), and width (w) = 2 × minor axis (b). The surface area (S) and volume (V) were calculated using the following equations:

S=4πb(sin45°(a−b)+b) (2)

$$V=\frac{4}{3}\pi ab^{2}$$ (3)

The cell wall thickness was determined using transmission electron microscopy (TEM) (JEOL-1230, JEOL Ltd. Japan). Four types of active yeast cells were used to prepare the specimens for TEM. Thirty cells were randomly selected from five different visions. For each cell, four different points were measured. The cell wall thickness statistics were obtained using a frequency histogram.

### Chemical composition of cell wall analysis

Active yeast cells were disrupted by shaking with glass beads [23], and the cell wall carbohydrates were hydrolyzed with 2 M H_2_SO_4_ at 100°C for 4 h according to the method of Dallies et al. [34]. For the extraction and purification of 1,3-β-glucan and 1,6-β-glucan, the cell wall fractions were extracted with 3% NaOH at 75°C for 6 h. The alkali-insoluble carbohydrates were then extracted with 0.5 M acetic acid at 90°C for several times. The alkali-insoluble and alkali-soluble glucan were the 1,3-β-glucan and 1,6-β-glucan respectively [35]. A Dionex Bio-LC system (ICS2500, USA) coupled with an ED 50 Electrochemical Detector was used for the quantitative analysis of the cell wall carbohydrates. Carbohydrates were analyzed by a Zorbax NH_2_ column (250 × 4.6 mm, 5 μm diameter, Agilent Technologies, USA) with a guard column (12.5 × 4.6 mm, 5 μm diameter, Agilent Technologies, USA). Deionized water: 0.5 mol/L NaOH (3.5: 96.5; v/v) was used as the isocratic mobile phase with a flow rate of 1 mL/min at room temperature (30°C). Chitin content was determined using an enzymatic method, and the released glucosamine was quantified as described in other reports [36].

### Patulin desorption assay

Four types of inactivated yeast cells were tested, desorption assay was conducted after patulin adsorption test. Firstly, inactivated yeast cells (3× 10^7^ per milliliter) were suspended in 1 mL of 200 μg/L patulin solution in a shaker incubator (150rpm at room temperature for 20 h), after patulin adsorption, the supernatants and sediments were separated by centrifugation at 3600 × *g* for 5 min. Supernatants were used to detecting patulin remaining amount, the sediments were then collected to conduct patulin desorption assay with 1 mL of ethyl acetate in a shaker incubator (150rpm at room temperature for 3h, 6h, 9h, 12h, 18h and 24h). Samples were taken out at different time points and dried by a rotary evaporator (RE-5205, Shanghai Yarong Biochemistry Instrument Factory, China) at 40°C. Finally, the detection assay was conducted after patulin was re-dissolved in 1 mL of 0.5% acetic acid solution (pH 4.0). The patulin desorption efficiency (*X* %) was calculated using the following equation:

$$X(\%)=\frac{X_{1}}{1-X_{2}}\times 100\%$$ (4)

where *X*_*1*_ is patulin re-dissolving concentration after patulin desorption (μg/L), *X*_*2*_ is patulin remaining concentration in supernatants after patulin adsorption (μg/L).

### Patulin adsorption in commercial kiwi fruit juice

Commercial kiwi fruit juice was diatomite filtered and sterilized before patulin adsorption. Inactivated yeast cells (100mg) were added into 10mL of 200μg/L patulin-contaminated commercial kiwi fruit juice in conical flasks. The control (without patulin addition) and test samples were incubated at 150 rpm at room temperature for different times (3h, 6h, 9h, 12h, 18h, 24h). There were three replicates for each strain. After adsorption, the sludge was separated by centrifugation at 3600 × *g* for 10 min. The treated juices were collected for patulin extraction and detection [37].

### Statistics analysis of data

All of the experiments were conducted three times to obtain the final results, and each independent experiment was generally performed in triplicates, the data were presented as mean ± standard deviation. Data were subjected to one-way ANOVA using Statistical Analysis System (SAS Inst., Cary, N.C., U.S.A.). Data on covariance, pearson correlation, Sig. 2 (tailed) and sum of squares were analyzed by Pearson Correlation Analysis using SPSS Statistics (SPSS version 17.0 for Windows, 2009). *P* values less than 0.05 were considered statistically significant.

## Results

### Effect of cell types on patulin adsorption

The patulin adsorption ratios were compared with four different species of yeast cells for four different treatments respectively (Table 1). In the experiment, different treatments of cells made their cell wall morphologies different, that active and inactivated cells represented cell wall integrity, while protoplasts and destroyed cells represented cell wall deficiency and cell wall damage respectively. The results of Table 1 indicated that patulin adsorption ratios were mainly affected by yeast species and yeast cell wall integrity. The adsorption ratios of the active and inactive cells ranged from 71.4%−88.7% by different yeast cells. All cells have patulin adsorption capability, and no significant differences were found between the active and inactive cells from the same strain. These results verified that patulin adsorption was more likely caused by cell surface adsorption than complex enzyme reactions. In addition, results of the Table 1 demonstrate an influence of the yeast specie in mycotoxins adsorption, in particular among the integrated cells (active and inactivated cells). *Pichia anomala* (B-2p) has a lowest affinity for patulin than *Candida tropicalis* (N-10) and the two *Saccharomyces cerevisiae* strains (WLS38 and 7#).

**Table 1:** Comparison of patulin adsorption ratio using different treatments.

Strains	Different treatments of yeast cells^#^			
	Active cells (%)	Inactive cells (%)	Protoplasts (%)	Destroyed cells (%)
**N-10**	85.1 ± 1.16a*	88.7 ± 0.95a	11.70 ± 0.94	44.76 ± 1.62
**7#**	80.3 ± 1.00b	78.9 ± 2.10b	10.22 ± 0.40	45.21 ± 6.05
**WLS-38**	80.9 ± 1.20b	81.7 ± 1.30b	10.60 ± 0.64	43.44 ± 3.86
**B-2p**	71.6 ± 0.63c	71.4 ± 1.40c	11.46 ± 0.54	44.86 ± 3.97

^#^ Inactive cells were treated with autoclaving; Protoplasts were treated with snail enzyme; Destroyed cells were treated with ethyl alcohol.

* Different letters indicate significant differences according to Duncan's new multiple range method (*p* < 0.05).

**Fig 2:**
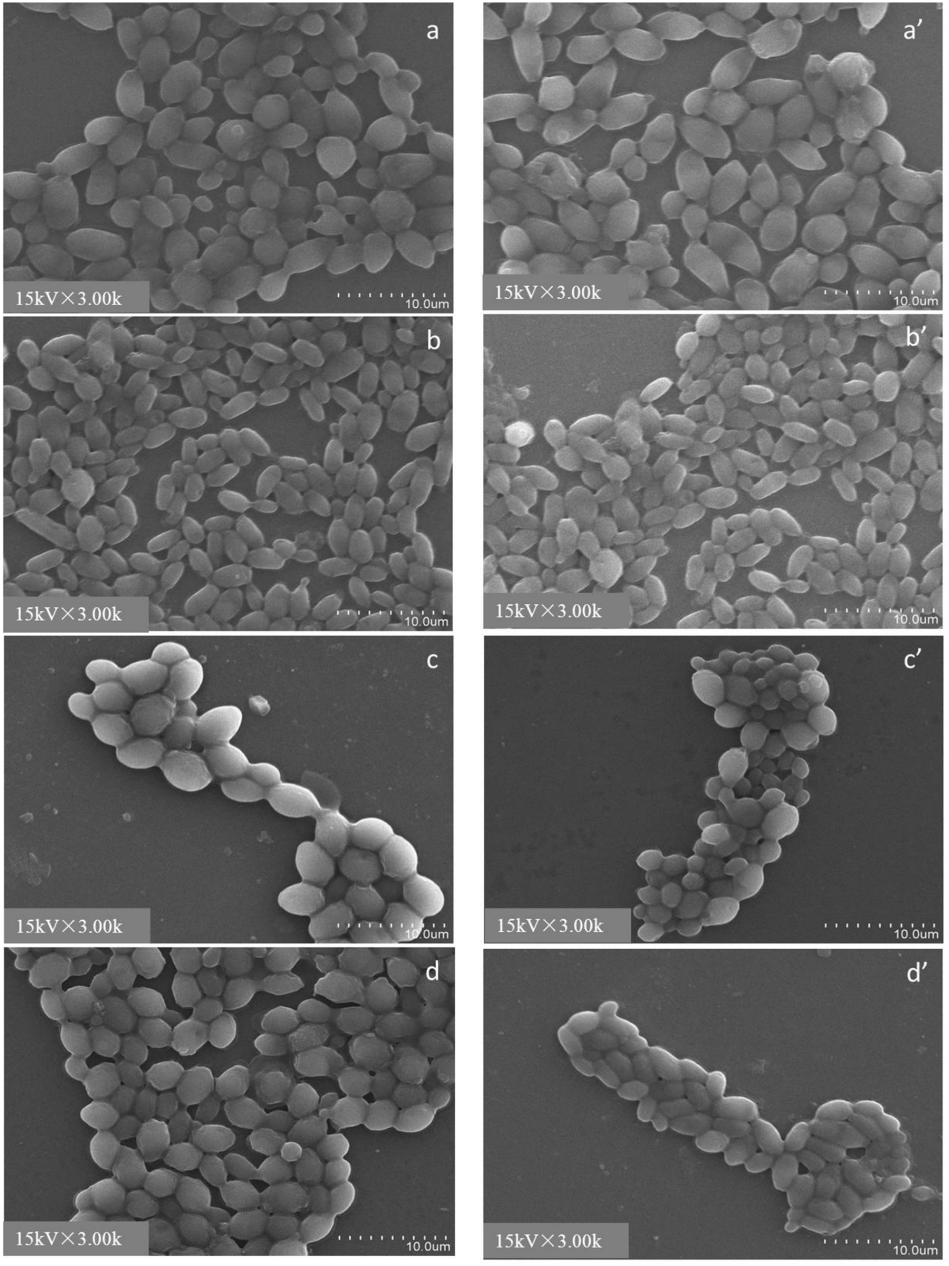
SEM images of activated cells and heated inactivated cells at 3,000 × magnification. a, b, c, d are activated cells of N-10, B-2p, 7# and WLS-38, respectively; a′, b′, c′, d′ are heated inactivated cells of N-10, B-2p, 7# and WLS-38, respectively.

**Fig 3:**
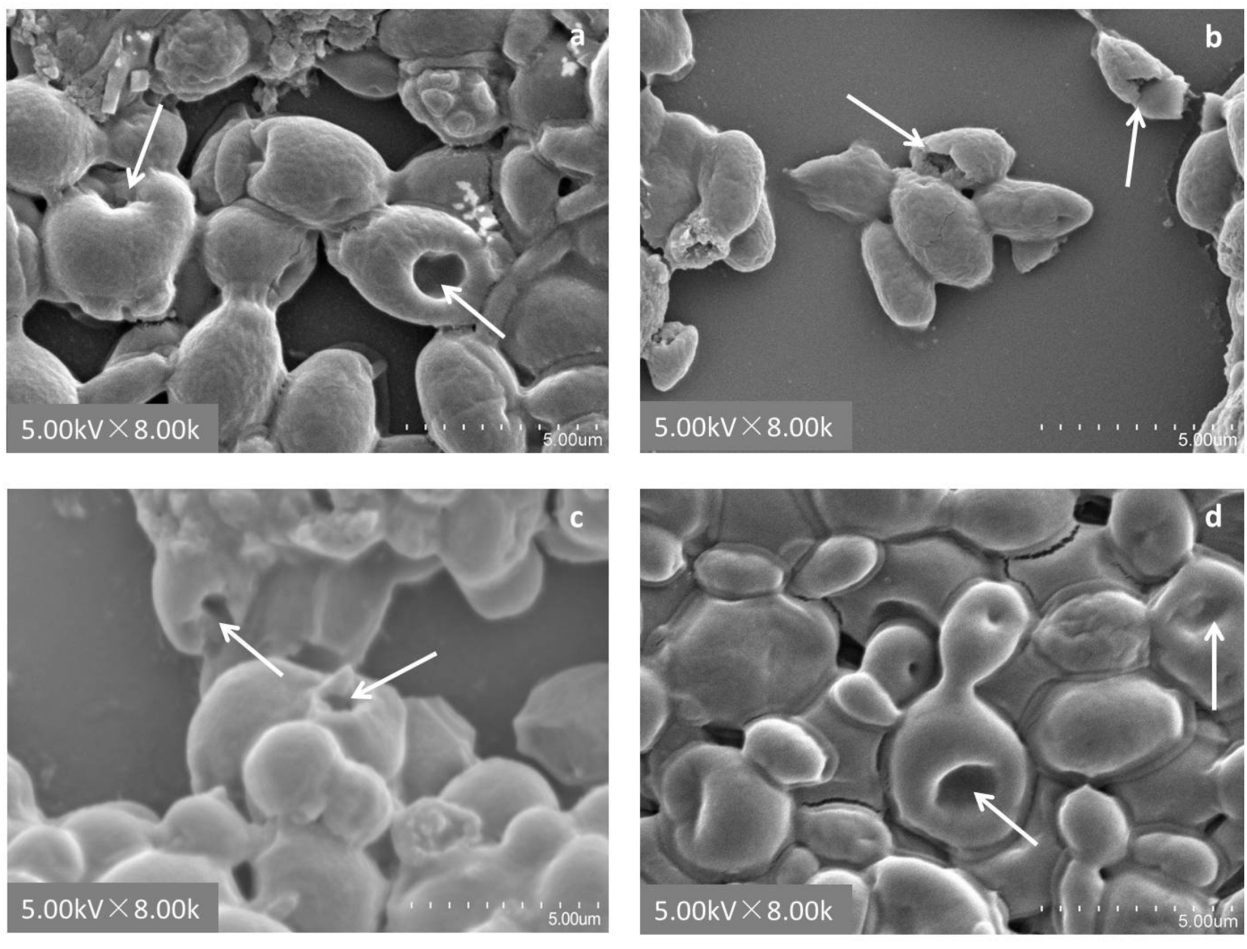
SEM images of yeast cells treated with ethyl alcohol for 6 h at 8,000 × magnification. (a) N-10, (b) B-2p, (c) 7#, and (d) WLS-38. Arrowhead ‘→’ means cells were regional invaginations.

The morphologies of the cells were not affected by heated treatment, the cells maintained plump and flat as active cells after heated inactivation treatment with autoclaving (Fig 2). The patulin adsorption capability was significantly reduced by the destroyed cells. The adsorption ratio of all strains decreased to 45% after ethyl alcohol treatment. The SEM image (8,000 ×) (Fig 3) shows that most cells have regional invaginations, and that overall shrinkage was observed after the cells were treated for 6 h with ethyl alcohol compared with their original morphology (3,000 ×). The protoplasts of the four strains almost lost their patulin adsorption capabilities because of the losing of their cell walls with the action of snail enzyme. The above results demonstrated that the yeast cell wall is important for the patulin adsorption process. The adsorption ratios of the yeast cells were decreased sharply by damaging or losing their cell walls.

### Effect of yeast cell volume and surface on patulin adsorption

The SEM images of the four yeast strains are shown in Fig 2a, 2b, 2c and 2d. The images show that all yeast strains were ellipsoid. The N-10 strain was nearly circular, and B-2p was slightly smaller and flatter than *S*. *cerevisiae* 7# and WLS-38. To measure the length and width of the cells, fifty cells from five different visions were randomly selected. The measurements, and cell volume and surface area calculation results are listed in Table 2. The results showed that the length and width of *C*. *tropicalis* N-10 and *S*. *cerevisiae* were significantly similar compared with each other. In addition, the differences among their cell volumes and surface areas were not significant. The *P*. *anomala* B-2p strain, which had the weakest patulin adsorption capability, also had smaller length and width. The cell volume and surface area were also smaller by about a third compared with the other strains.

**Table 2:** Yeast cell micromorphology and cell wall thickness calculation^#^.

Strain	length × width(μm)	surface area (μm^2^)	cell volume(μm^3^)	cell wall thickness (nm)
**N-10**	(3.58 ± 0.13) × (2.63 ± 0.07)	27.21 ± 1.23a*	12.97 ± 0.88a	158.47 ± 4.17a
**7** #	(3.55 ± 0.04) × (2.64 ± 0.04)	27.12 ± 0.59a	12.91 ± 0.44a	102.12 ± 3.38b
**WLS-38**	(3.52 ± 0.05) × (2.55 ± 0.09)	25.83 ± 1.23b	11.97 ± 0.92b	103.55 ± 2.88b
**B-2p**	(3.29 ± 0.16) × (2.06 ± 0.09)	18.94 ± 1.38c	7.35 ± 0.81c	61.83 ± 2.07c

^#^ Length and width of yeast cell data were abtained by measuring large number of cells from their SEM photographs; Cell surface areas and cell volume data were calculated with Eqs (2) and (3) according to the major and minor axises (a and b) measurement; Data of Cell wall thicknesses was abtained by the results of the frequency histogram in Fig 5.

* Different letters indicate significant differences according to Duncan's new multiple range method (p < 0.05).

### Effect of cell wall thickness on patulin adsorption

**Fig 4:**
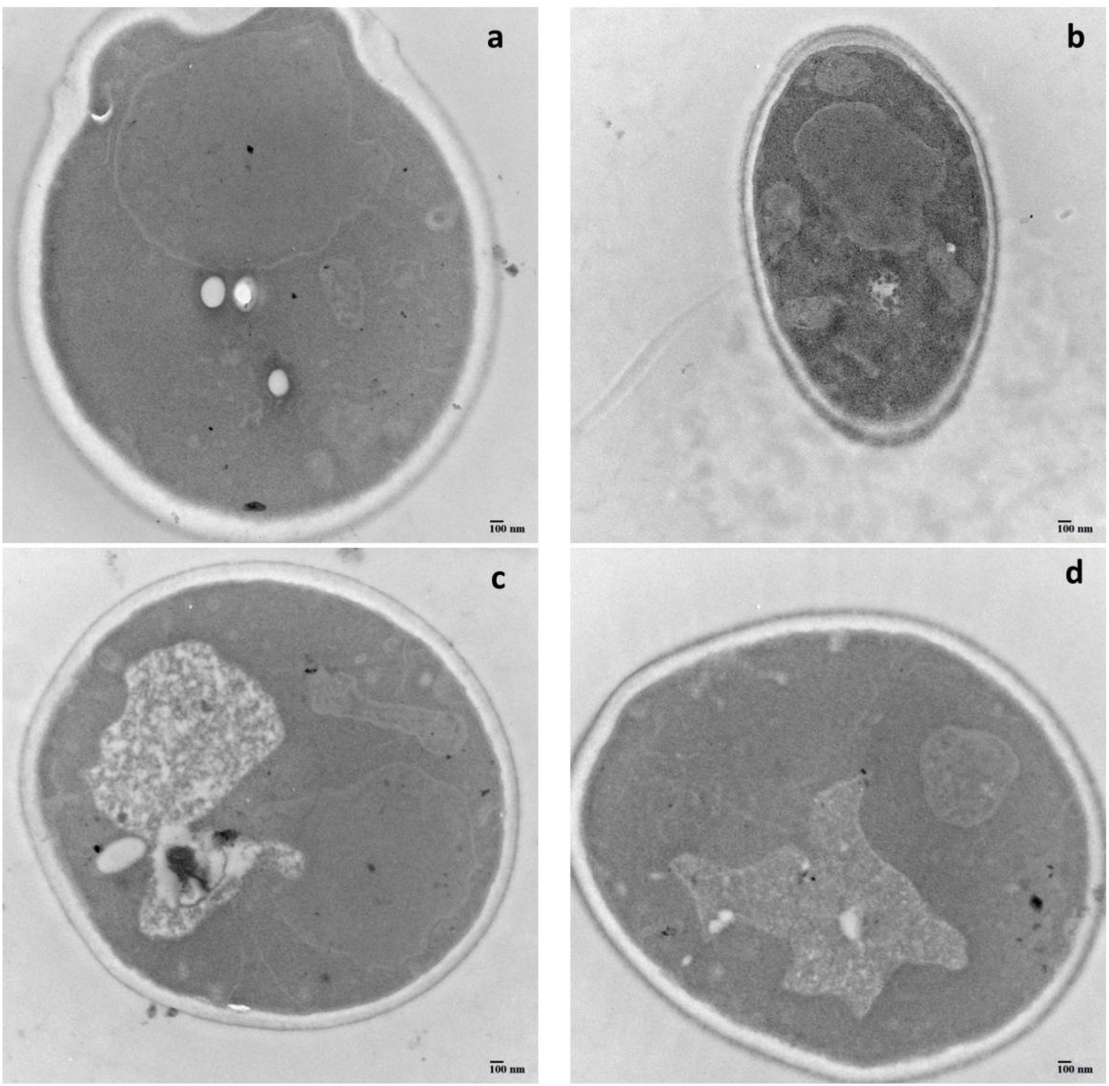
TEM images of four types of active yeast cells at 25,000 × magnification. (a) N-10, (b) B-2p, (c) 7#, and (d) WLS-38.

**Fig 5:**
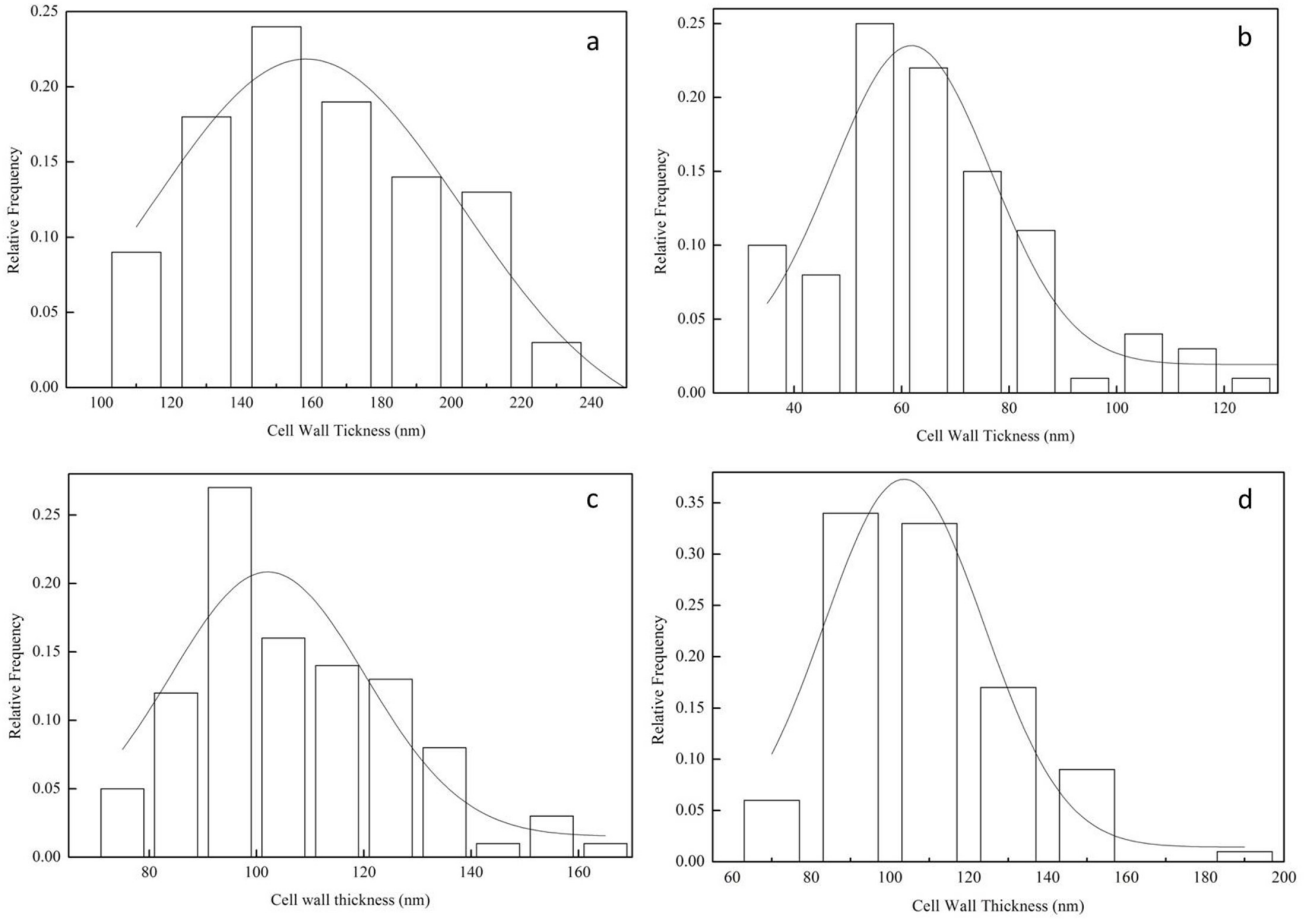
Histogram statistical results of yeast cell wall thickness. (a) N-10, (b) B-2p, (c) 7#, and (d) WLS-38.

The ultra-structures of the four yeast cells are shown in the TEM images at 25,000 × magnifications (Fig 4), and the cell wall thickness measurements are calculated using the frequency histogram (Fig 5), the statistical data are listed in Table 2. Fig 4 showed the bi-layered structure of the cell wall. Indeed, it appears in image b of B-2p that the inner layer was the thinnest, and has a similar thickness than the outer layer, as compared to other strains species. The inner layer of strain N-10 was symmetric and thick, with the thickness of 158 nm, was twice more than that of the thinnest cell wall of the strain B-2p. The inner layer thickness of 7# and WLS-38 were moderate at around 102 and 103 nm, respectively. The inner layer is mostly constituted of chitin, β-1,3-glucans and β-1,6-glucans, while the outer layer is made of mannoproteins. Thus, the content of the cell wall polysaccharides was measured and discussed in this study.

### Effect of 1,3-β-glucan on yeast cell wall thickness and patulin adsorption

**Fig 6:**
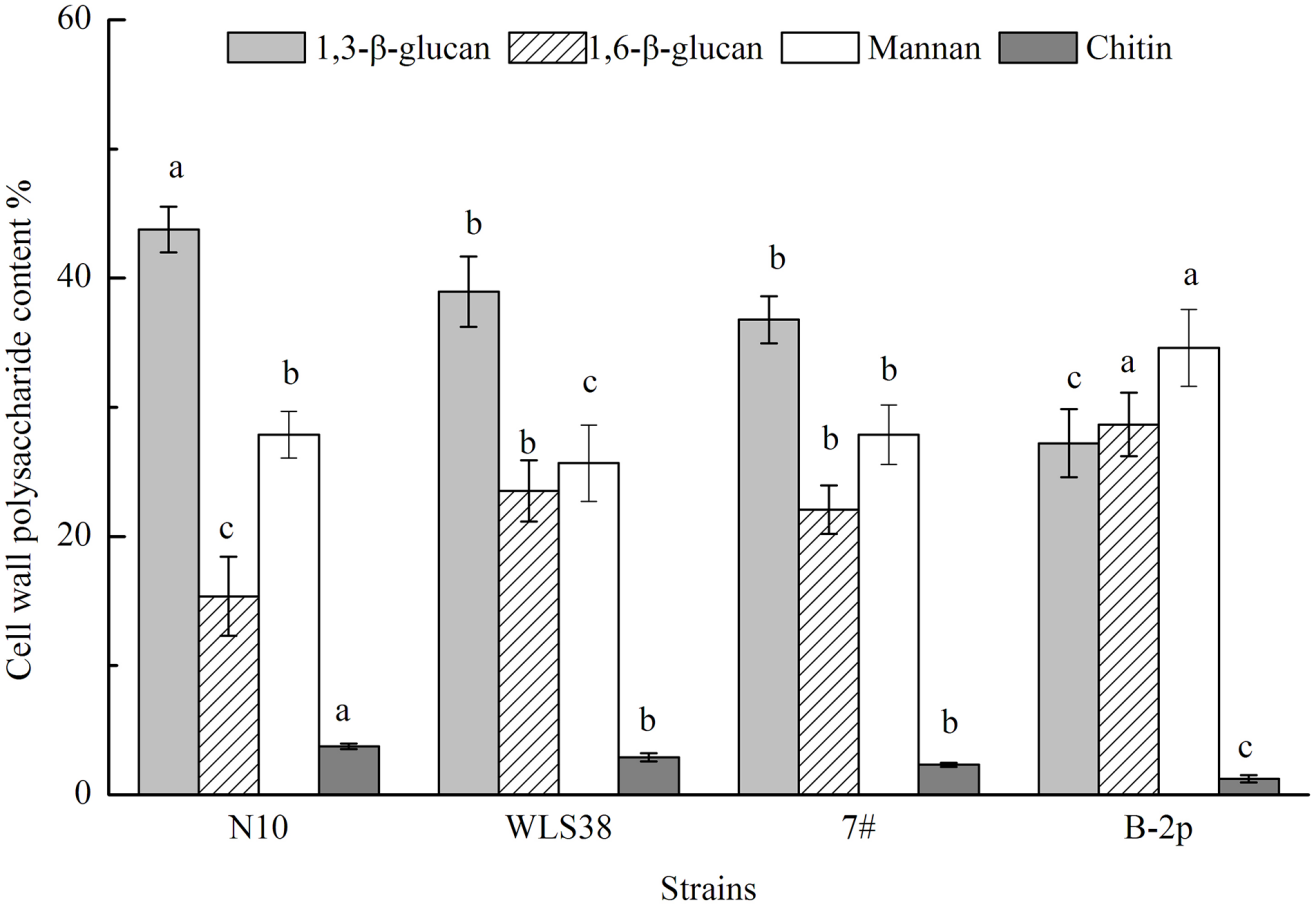
Cell wall polysaccharide contents of four active yeast strains. Bars with different letters are significantly different (*p* < 0.05).

The dry weights of the cell wall fractions of N-10, 7#, WLS-38, and B-2p were 35.93%, 30.77%, 31.63%, and 27.23% of the total cells, respectively. The cell wall polysaccharides were mainly composed of 1,3-β-glucan, 1,6-β-glucan, mannan, and minor chitin, and their contents are shown in Fig 6. Different cells had different proportions of polysaccharide compositions, indicating notable interspecific differences and non-significant intraspecific differences. However, in the same species, *S*. *cerevisiae* WLS-38 and 7# indeed differ on their polysaccharide compositions. The proportions of 1,3-β-glucan and 1,6-β-glucan were 38.9% and 23.5%, 36.8% and 22% in *S*. *cerevisiae* WLS-38 and 7#, respectively. The 1,3-β-glucans and 1,6-β-glucans consisted about 50%-60% of the polysaccharide composition, The N-10 had the highest 1,3-β-glucan content, but presented the least amount of 1,6-β-glucan. In The chitin content had a similar trend with the 1,3-β-glucans. The mannan contents of different strains were significantly similar compared with each other.

Table 3 shows that the results of the correlational analysis of the cell wall characteristics (thickness and 1,3-β-glucan content) and patulin adsorption were significant at 0.05 level. A high 1,3-β-glucan content was detected in N-10, which showed the thickest cell wall and highest patulin adsorption capability. The 1,3-β-glucan content of strains 7# and WLS-38 were followed by N-10, which had moderate cell wall thickness and patulin adsorption capability. The B-2p strain, which had the lowest 1,3-β-glucan content, presented the thinnest cell wall and weakest patulin adsorption capability.

**Table 3:** Correlational analysis of patulin adsorption with cell micromorphology and cell wall.

Parameter	Pearson Correlation	Sig. (2-tailed)	Sum of Squares and Cross-products	Covariance
**Cell volume**	0.834	0.166	47.867	15.956
**Cell surface areas**	0.834	0.166	70.580	23.527
**Cell wall thickness**	0.981	0.019*	836.109	278.703
**1,3-β-glucan content**	0.984	0.016*	146.527	48.842

* Correlation is significant at 0.05 level.

**Fig 7:**
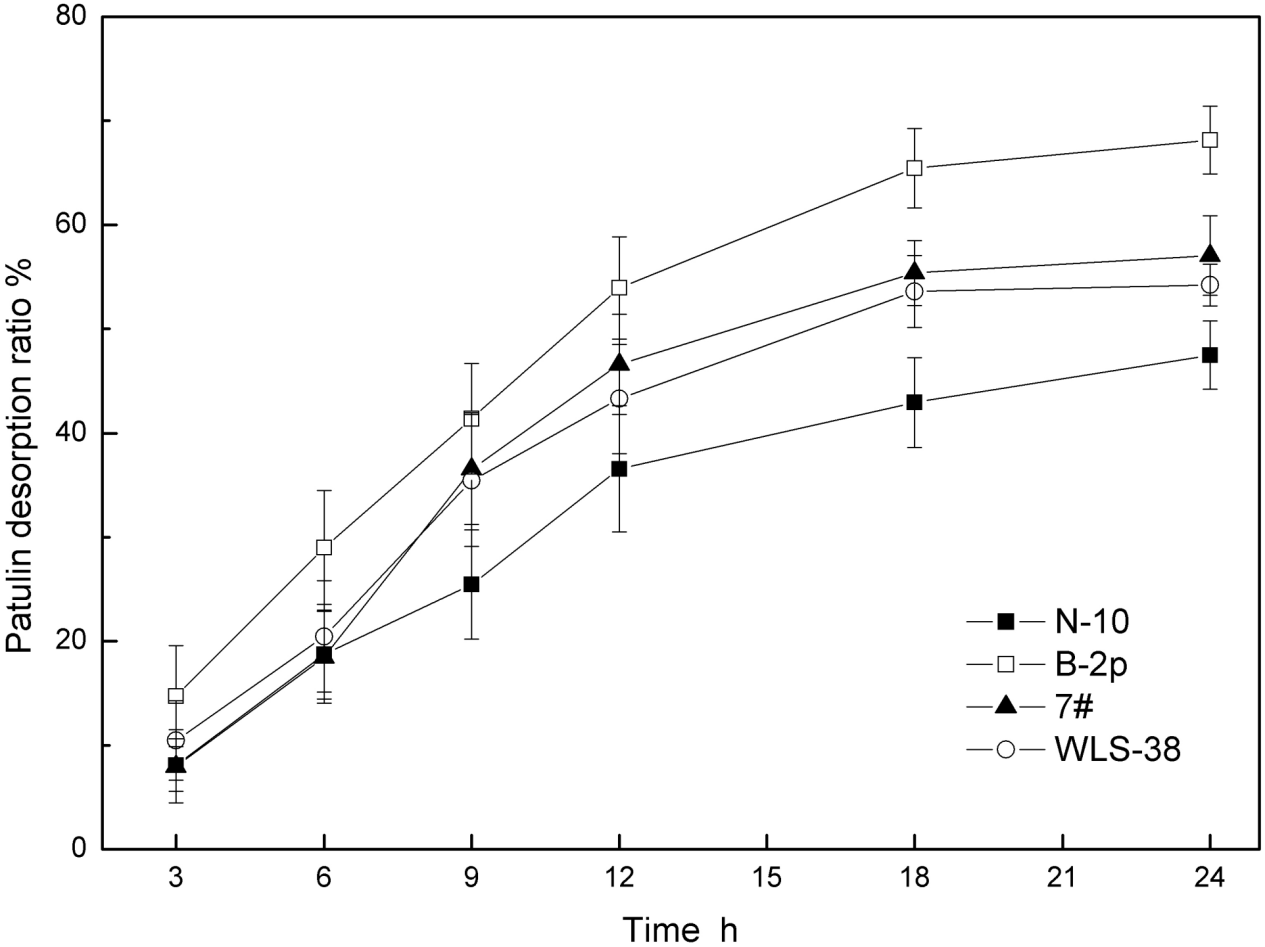
Patulin desorption of four inactivated yeast cells in ethyl acetate.

Patulin desorption results is shown in Fig 7, four yeast cells presented different patulin desorption capabilities after they binding the same amount of patulin. B-2p cells had a highest patulin desorption capability during 18–24 h, with 68.2%, which owned the lowest 1,3-β-glucan content, thinnest cell wall and weakest patulin adsorption capability. Patulin desorption capability in a descending order was: B-2p > 7# > WLS-38 > N-10. It showed that a thinner cell wall structure which has less 1,3-β-glucan, has less interstices for patulin to hide while easy for escaping; a thicker cell wall structure which has more 1,3-β-glucan, has more interstices for patulin to hide while difficulty for escaping. Thus, there were more patulin escaped from a thinner cell wall like B-2p strain.

### Commercial kiwi fruit juice adsorption

**Fig 8:**
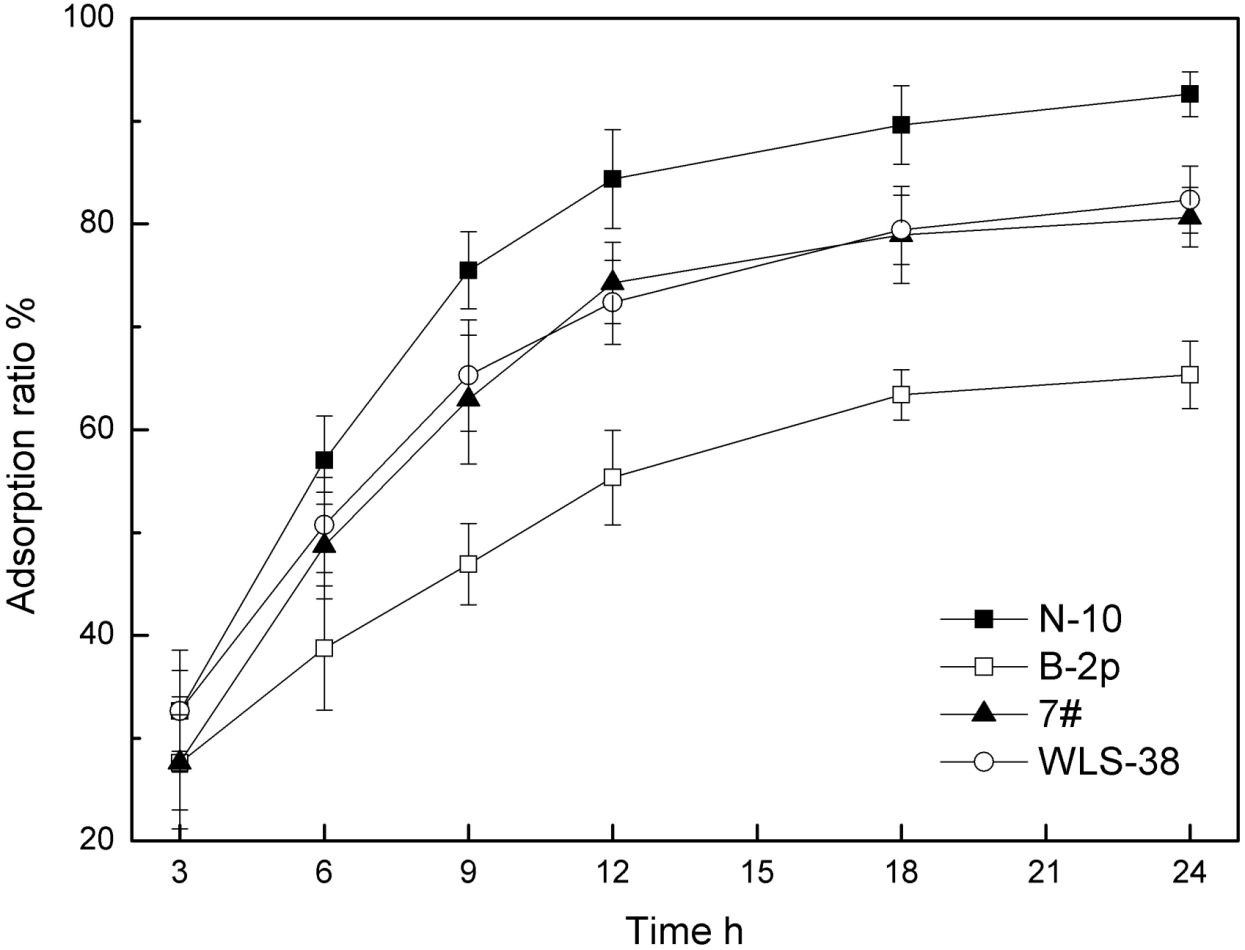
Patulin adsorption in commercial kiwi fruit juice by four inactivated yeast cells.

The results of patulin adsorption in kiwi fruit juice are shown in Fig 8. All the tested cells could bind patulin from kiwi fruit juice efficiently, and the adsorption ratio was ranged from 65.3% to 92.6%. Patulin adsorption ratio increased with the reaction time increasing, the adsorption process reached equilibrium at 18–24 h. N-10 cells had the highest patulin adsorption capability, while B-2p cells were the weakest one, the patulin adsorption capability were consistent with the test in acidic aqueous solution. With 100 mg inactive yeast cells added, patulin level in 10 mL contaminated kiwi fruit juice could be reduced from original concentration 200 μg/L to under the inspection qualification with N-10 strain. Therefore, the yeast cells could be used as the efficient adsorbents for patulin adsorption in commercial kiwi fruit juice.

## Discussion

The cell wall has been identified as the main site for mycotoxin adsorption using microbial cells [29]. The study focused on explaining the patulin adsorption behavior based on the cell wall surface morphology and composition. Four yeast strains were used (*C*. *tropicalis* N-10, *S*. *cerevisiae* 7# and WLS-38, and *Pichia anomala kurtzman* B-2p) to study the patulin adsorption behavior. The micromorphology of each cell, including the cell volume and surface area, as well as the physical structure and chemical compositions of the cell wall, were observed and investigated.

In this study, the patulin adsorption ratio of the active cells was significantly similar to that of the inactive cells, suggesting that the cell and enzymatic activities nearly had no impact on the patulin adsorption. The patulin adsorption more likely occurs through surface adsorption than metabolism or enzymatic reaction. This result was consistent with that of other studies [18, 24]. The patulin adsorption capability dramatically decreased when the cell wall presented an overall shrinkage and regional invaginations. This finding implied that the cell wall morphology and integrity are important for patulin adsorption. By treating the cells with snail enzyme, the patulin adsorption capability was nearly lost when the cell wall was disrupted, indicating that the cell surface was the main region of patulin adsorption. The cell wall may have an important role in patulin adsorption.

A significant difference was found in the patulin adsorption among the different species; however, no evident difference was observed within the same species (Table 1). Similar results have been previously reported by Hatab [13]. This phenomenon might be attributed to the different cell morphologies or cell wall compositions, because the yeast cell wall composition varied with the species [23]. The results show that B-2p strain showed the weakest patulin adsorption capability, and had the smallest cell volume and cell surface area. However, the relationship between the cell morphology and patulin adsorption ratio was not significantly similar with those of the other strains. Moreover, the results of correlational analysis for the patulin adsorption capability and cell morphology show no significant relationship, whether for the cell volume or surface area (Table 2). The results demonstrated that the cell morphology was one of the influential factors for patulin adsorption, but not necessarily the key factor.

Cell wall, serving as the first barrier against exposure to patulin, and its composition and physical structure should be taken into account. Cell wall is an elastic three-dimensional network, consisting of insoluble 1,3-β-glucan and small amounts of chitin that forms the main structural frame. The insoluble 1,3-β-glucan content influences the density and thickness of the network structure [38]. The results of this study showed that the 1,3-β-glucan content was well correlated with the cell wall thickness and patulin adsorption capability. The N-10 strain, which had the highest 1,3-β-glucan content, had the thickest cell wall and best patulin adsorption capability, indicating that a higher 1,3-β-glucan content could result in a denser and thicker three-dimensional network. Thus, more meshes were available to adsorb patulin molecules. The synthetic ability of the 1,3-β-glucan varied with the progression of the cell cycle, and the proteins in the cross-linking cell wall depended on the yeast strain [38]. The three-dimensional network formed by 1,3-β-glucan was different among the strains, resulting in different patulin adsorption capabilities. The patulin adsorption capability of a strain could dramatically decrease or may disappear when the network is artificially damaged or removed. The network formed by 1,3-β-glucan was very important for the integrity of the cell wall structure and patulin adsorption capability. However, the same specie of the two *S*. *cerevisiae* strains WLS38 and 7#, they have similar cell wall thickness, different 1,3-β and 1,6-β-glucan content, have different percentage of patulin adsorption, this may be demonstrated by a fact that a role of 1,6-β-glucan to stabilize the role of toxin-glucan interactions [28]. Patulin desorption assay indicated that the content of 1,3-β-glucan was a critical factor for yeast cell wall, to the extent that important on their patulin adsorption. The higher of cell wall thickness, thus the higher of patulin adsorption capability, the weaker of patulin desorption capability on the contrary.

## Conclusion

The patulin adsorption capability of the yeast cell wall was mainly determined by the insoluble 1,3-β-glucan content, which forms the backbone of the network and thus determine the thickness of the yeast cell wall. Therefore, 1,3-β-glucan is an important factor that affects the patulin adsorption capability of yeast. The adsorption process could be considered as the embedment of a free patulin into a three-dimensional network structure. The patulin adsorption capability increased with increasing network density. However, to validate our hypothesis, a better methodology combining yeast cell wall network structure with patulin adsorption capability is needed. Nevertheless, this study provided experimental foundation and theoretical basis for promoting research on patulin adsorption. Moreover, juice adsorption assay indicated that inactive yeast cells could be successfully used as patulin adsorbents in kiwi fruit products.
